# Comparing New-Generation Candidate Vaccines against Human Orthopoxvirus Infections

**Published:** 2017

**Authors:** R. A. Maksyutov, S. N. Yakubitskyi, I. V. Kolosova, S. N. Shchelkunov

**Affiliations:** State Research Center of Virology and Biotechnology «Vector», Koltsovo, Novosibirsk region, 630559 , Russia; Institute of Cytology and Genetics, Siberian Branch of Russian Academy of Sciences, Novosibirsk, 630090 , Russia

**Keywords:** DNA vaccine, vaccinia virus, virulence genes, protective potential, smallpox

## Abstract

The lack of immunity to the variola virus in the population, increasingly more frequent cases of human orthopoxvirus infection, and increased risk of the use of the variola virus (VARV) as a bioterrorism agent call for the development of modern, safe vaccines against orthopoxvirus infections. We previously developed a polyvalent DNA vaccine based on five VARV antigens and an attenuated variant of the vaccinia virus (VACV) with targeted deletion of six genes (VACΔ6). Independent experiments demonstrated that triple immunization with a DNA vaccine and double immunization with VACΔ6 provide protection to mice against a lethal dose (10 LD_50_) of the ectromelia virus (ECTV), which is highly pathogenic for mice. The present work was aimed at comparing the immunity to smallpox generated by various immunization protocols using the DNA vaccine and VACΔ6. It has been established that immunization of mice with a polyvalent DNA vaccine, followed by boosting with recombinant VACΔ6, as well as double immunization with VACΔ6, induces production of VACV-neutralizing antibodies and provides protection to mice against a 150 LD_50_ dose of ECTV. The proposed immunization protocols can be used to develop safe vaccination strategies against smallpox and other human orthopoxvirus infections.

## INTRODUCTION

The *Orthopoxvirus *genus of the Poxviridae family includes human-pathogenic species, such as the variola virus (VARV), monkeypox virus (MPXV), cowpox virus (CPXV), and vaccinia virus (VACV). Mass vaccination with a conventional VACV-based vaccine protects not only from VARV, but also from the closely related MPXV and CPXV [1]. After 1980, the share of the population sensitive to VARV and other orthopoxviruses pathogenic to humans has constantly increased due to the eradication of smallpox and cessation of widespread immunization against the disease. This is evidenced in the increasingly more frequent multiple cases of orthopoxvirus infections in humans caused by such viruses as MPXV, CPXV, and VACV [2-6]. Moreover, VARV is considered a potential agent of bioterrorist attacks, which could have catastrophic consequences for the entire world population [6]. The lack of effective antiviral drugs and the risk associated with conventional VACV-based live vaccines, because of severe postvaccinal complications, necessitate the development of modern, safe orthopoxvirus vaccines and protocols for their use [7, 8].

Earlier, we developed a recombinant variant VACΔ6 with targeted knockdown of six genes, encoding hemagglutinin *(A56R), *the gamma-interferon-binding protein *(B8R), *thymidine kinase *(J2R), *the complement-fixing protein *(C3L), *the Bcl2-like apoptosis inhibitor *(N1L), *and the *A35R *gene, which controls antigen presentation by the class II major histocompatibility complex (MNSII), based on the LIVP VACV strain used in the Russian Federation for the vaccination of humans. It has been shown that inactivation of selected virulence genes does not affect the reproductive properties of VACV in mammalian cell cultures. The VACΔ6 strain is significantly less reactogenic and neurovirulent and more immunogenic compared to the parent LIVP strain. Double subcutaneous injection of recom binant variant VACΔ6 induces significantly higher levels of virus-neutralizing antibodies in mice than the parental LIVP strain and provides complete protection to mice against the highly pathogenic ectromelia virus (ECTV), as opposed to the effect of the LIVP strain in this model, which is approved as a smallpox vaccine [9, 10].

Earlier, we implemented another independent approach to vaccinal prevention of smallpox. We developed a polyvalent DNA vaccine based on a mixture of recombinant plasmids containing the genes of five virion proteins of the VARV: A30, F8, M1, which are constituents of the surface membrane of intracellular virions, and A36, B7, which are located on the membrane of the extracellular form of the virus, under the control of the CMV promoter. Triple intradermal immunization with a polyvalent DNA vaccine induced the production of virus-neutralizing antibodies and provided complete protection to mice against ECTV infection at a dose of 10 LD_50_ [11-13].

Along with the development of fundamentally new vaccines, a combination of various types of vaccines which can complement each other and induce strong and broad immunity is another promising avenue in improving the efficacy of smallpox vaccination [14]. Such a heterologous immunization strategy (prime-boost), where the subunit vaccine (DNA vaccine) is used to prime the immune system and where the attenuated variant of VACV is used for subsequent booster vaccination, is considered promising.

This study compared immunity against smallpox induced by double immunization with various combinations of polyvalent DNA vaccines and a highly attenuated VACΔ6 strain.

## EXPERIMENTAL

**Bacteria, viruses, cell cultures**

In this study, we used *Escherichia coli XL2-blue, *the VACΔ6 strain [10], the LIVP VACV strain (derived from a Lister strain obtained from the Institute of Viral Preparations, Moscow), and a K-1 ECTV strain from the collection of SRC VB “Vector,” continuous cell culture 4647 of African green monkey kidney cells [15] from the collection of cell cultures of SRC VB “Vector” cultivated on a DMEM medium supplemented with 10% fetal bovine serum.

**Polyvalent DNA vaccine**

A set of recombinant plasmids based on the vector plasmid pcDNA3.1, bearing genes of five VARV antigens, including A30, F8, M1 antigens of the surface membrane of intracellular virions and A36, B7 antigens of the membrane of extracellular forms of the virus under the control of a cytomegalovirus promoter, was obtained previously [11-13]. Preparative quantities of plasmid DNA were accumulated in *E. coli *cells and purified using the EndoFree Plasmid Giga Kit (Qiagen, USA) according to the manufacturer’s recommendations. Plasmid DNA concentration was measured spectrophotometrically on a Ultrospec 3000 pro instrument (GE Healthcare Life Sciences, USA).

**Accumulation and purification of viruses**

A monolayer of 4647 cells grown in culture flasks with a growth surface of 175 cm^2^ (volume of 650 ml) was infected with VACV (VACΔ6 or LIVP strain), and the multiplicity of infection was 1 PFU/cell. The virus was incubated in a DMEM medium with 2% fetal bovine serum for 48 hours at 37°C until complete cytopathic effect, followed by the obtaining of a cryolysate (three freezing-thawing cycles) of the infected cells, and double or triple sonication of the latter in the 22 kHz MSE 500 disintegrator for 10–15 seconds. Cell debris was removed by low-speed centrifugation (10 min at 4,000 *g). *The supernatant was centrifuged for 1.5 hours at 30,000 *g. *The precipitated virus was re-suspended in 4 ml of saline. Infectious virus titer was determined using the agar-free plaque technique in a 4647 cell monolayer.

**Study of the immunogenicity and protectivity**

In this study we used Balb/c mice (females, weight 14–16 g, 5–6 weeks old) from the mouse bank of the SRC VB “Vector.” Mice were divided into groups of 10 animals. They were immunized with a polyvalent DNA vaccine subcutaneously and with a mixture of pcDNA-A30, pcDNA-A36, pcDNA-M1, pcDNA-F8, and pcDNA-B7 plasmids (50 μg of each plasmid, a total dose of 250 μg/100 μl per mouse) intradermally. The mice were immunized subcutaneously with VACΔ6 or LIVP strain at a dose of 107 PFU/100 μl per mouse. Control group mice were injected with a volume equal to that of the saline that was used to prepare virus dilutions. Immunization was performed twice at an interval of 21 days as shown in *Table 1*.

Blood samples were collected from the retrobulbar venous plexus of pre-anesthetized mice 19 days after the second immunization, incubated at 4°C for 24 hours to form a fibrin clot, and centrifuged for 10 min at 5,000 *g. *Serum preparations from one group of animals were then pooled and heated at 56°C for 30 min. Titer of VACV-neutralizing antibodies was determined on a 4647 cell culture according to [16], using serial fivefold dilutions of sera, which were mixed with an LIVP strain of VACV at the working dilution of 50 PFU/well. The effectiveness of the neutralization was calculated with respect to the number of plaques in the sera-free wells as -lg of the highest serum dilution, which provides 50% neutralization of VACV.

The animals under mild ether anesthesia were subjected to intranasal inoculation with ECTV, which is highly pathogenic to mice, at a dose of 150 LD_50_/20 μl per mouse according to [17] 21 days after the second immunization. The mice were followed for 14 days, and the number of survived and dead mice was recorded.

**Data analysis**

The statistical significance of the experimental data was evaluated based on the Student’s t-test using the Origin Professional 8.1.10.86 software. The differences were considered statistically significant at *P* < 0.05 [18].

## RESULTS

**Fig. 1:**
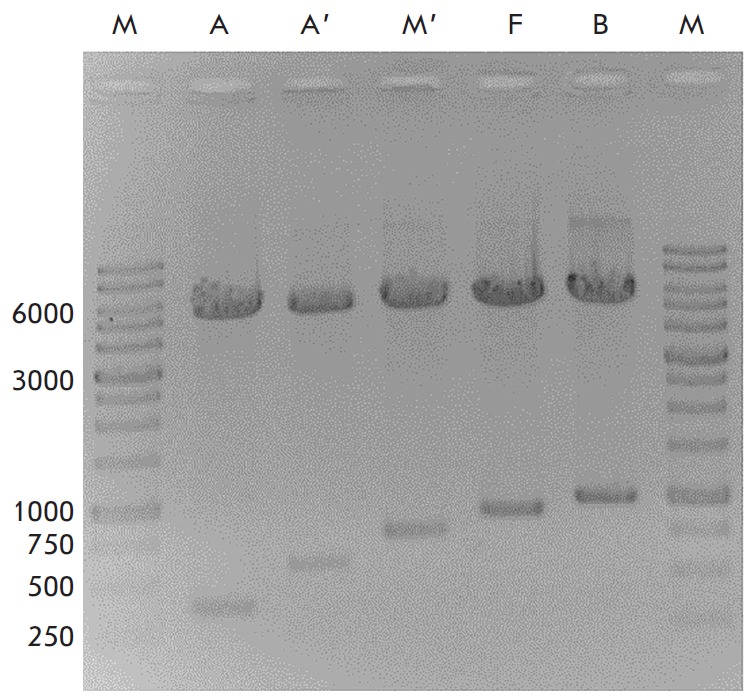
Result of electrophoretic separation of DNA fragments produced after hydrolysis of the recombinant plasmid with the restriction endonucleases *Asu*NHI and *Hin*dIII on a 1.2% agarose gel. A, A’, M’, F, B – DNA fragments obtained for the recombinant plasmids pcDNA-A30, pcDNA-A36, pcDNA-M1, pcDNA-F8, and pcDNA-B7, respectively*. *M – DNA ladder, fragment length in bp is shown on the left

Preparative quantities of pre-engineered pcDNA-A30, pcDNA-A36, pcDNA-M1, pcDNA-F8, and pcDNA-B7 plasmids were accumulated in *E. coli*cells and purified using the EndoFree Plasmid Giga Kit (Qiagen, USA) according to the manufacturer’s instructions, followed by confirmation of the accuracy of insertions by restriction analysis using AsuNHI and HindIII endonucleases *(Fig. 1)*and sequencing.

**Fig. 2:**
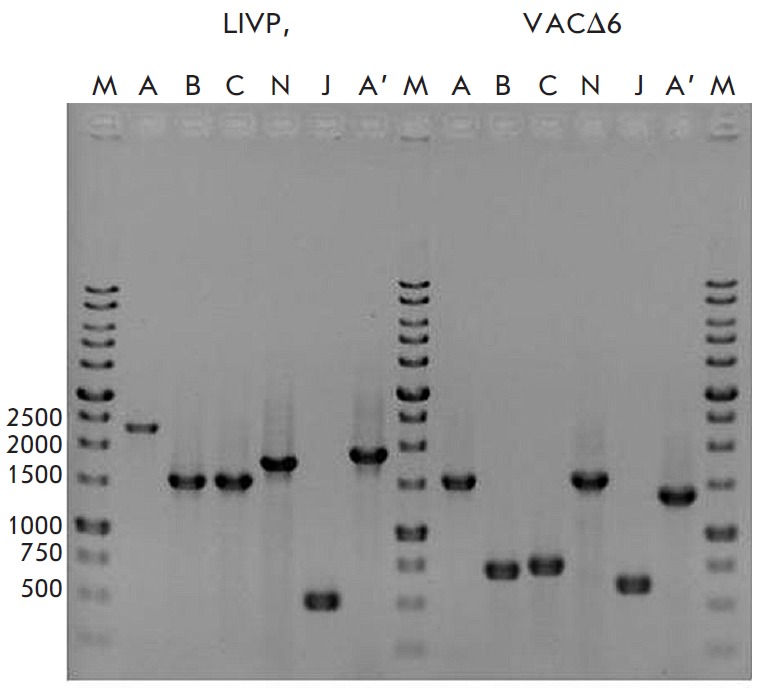
Verification of deletions/insertions by PCR. PCR products formed from DNA of the parent clone VACV LIVP and VACΔ6 with deletion of six virulence genes. A, B, C, N, J, A’ – PCR products obtained with the appropriate primer pairs for the *A56R, B8R, C3L, N1L, J2R, and A35R *genes*. *M – DNA ladder, fragment length in bp is shown on the left

VACΔ6 and LIVP vaccinia virus strains were produced in a 4647 cell culture recommended for the production of a smallpox vaccine [19] and purified according to the aforementioned method. The strains were identified using a PCR analysis based on the loci of six inactivated genes (*Table 2*, *Fig. 2*).

**Fig. 3:**
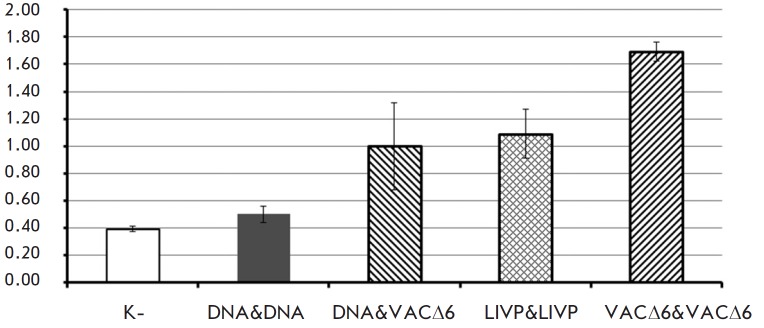
The level of serum-neutralizing activity against VACV, following double immunization with study preparations (DNA vaccine, VACΔ6 and LIVP VACV strains)

The immunogenicity of double immunization with various combinations (*Table 2*) of the polyvalent DNA vaccine and a highly attenuated strain VACΔ6 was assessed based on the level of induced virus-neutralizing antibodies in the mice serum sampled 21 days after the second immunization. As can be seen from the data shown in *Fig. 3*, the combination of DNA & VACΔ6 vaccines induced the accumulation of VACV-neutralizing antibodies whose level was comparable to the level of antibodies induced by double vaccination with a parent-strain LIVP. Moreover, double immunization with a VACΔ6 strain induced significantly higher levels of neutralizing antibodies, which is consistent with our previous results [10].

**Table 1:** Testing scheme to assess the immunogenicity and protection of the vaccines in animal experiments

Group	Vaccine, dose per animal		Protectivity test, day 42
	1^st^ immunization, day 1	2^nd^ immunization, day 21	
DNA&DNA	DNA vaccine 250 μg	DNA vaccine 250 μg	K-1 strain of ECTV, 150 LD_50_
DNA&VACΔ6	DNA vaccine 250 μg	VACΔ6 strain 10^7^ PFU	K-1 strain of ECTV, 150 LD_50_
VACΔ6&VACΔ6	VACΔ6 strain 10^7^ PFU	VACΔ6 strain 10^7^ PFU	K-1 strain of ECTV, 150 LD_50_
LIVP&LIVP	LIVP VACV strain 10^7^ PFU	LIVP VACV strain 10^7^ PFU	K-1 strain of ECTV, 150 LD_50_
K-	Saline	Saline	K-1 strain of ECTV, 150 LD_50_

**Fig. 4:**
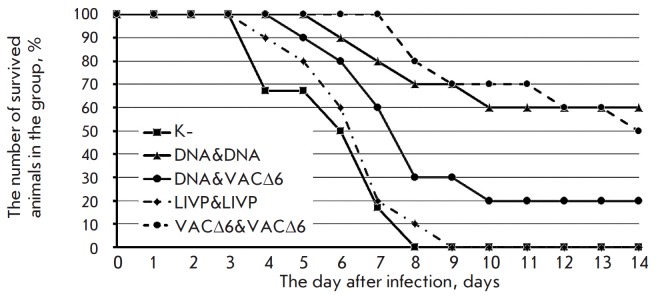
Time-course of mortality after double immunization of mice with the preparations under study(DNA vaccine, VACΔ6 and LIVP VACV strains), followed by challenge with ECTV at a dose of 150 LD_50_/mouse

As shown in our previous studies, triple immunization with a polyvalent DNA vaccine or double immunization with the VACΔ6 strain provides 100% protection to mice subsequently infected with ECTV at a dose of 10 LD_50_/mouse [10, 12]. For this reason, a significantly higher resolving dose of ECTV was used, 150 LD_50_/ mouse, in order to assess the differences in the effectiveness of the used immunization protocols. As a result, a partial protective effect of double immunization (DNA & VACΔ6, LIVP & LIVP and VACΔ6 & VACΔ6) was observed in three test groups (*Fig. 4*).

**Table 2:** PCR analysis aimed at identification of the recombinant VACV

Gene	Primer, nucleotide sequence (5’ → 3’)	LIVP strain, bp	VACΔ6 strain, bp
A56R	GTGGTATGGGACACCACAAATCCAA ATTAAACATTCCTAGAATTAATCCCGCTC	2366	1425
B8R	TCACAAATATGATGGTGATGAGCGA CGTGATATACCCTAGCCATAGGCAT	1555	737
C3L	TCGCGCTTTACATTCTCGAATCT TGTTCGTGTGTTCTTGCGGTGA	1542	751
N1L	GGGTTGGATCCTTTACACATAGATCTACTACAGGCGGAACA GGGAAAGCTTAATTTGTGAAGATGCCATGTACTACGCT	1784	1431
J2R	ATATGTTCTTCATGCCTAAACGA ATGAAGGAGCAAAAGGTTGTAAC	512	617
A35R	ACGACGGATGCTGAAGCGTGTTATA AAACGATGTTACCAATCGTTTGCTAGGT	1880	1360

Maximum survival was observed in the VACΔ6&VACΔ6 group animals, who received double vaccination with VACΔ6, and in DNA & VACΔ6 group animals, wherein the immune system was primed using the polyvalent DNA vaccine, and attenuated VACΔ6 was used for subsequent booster vaccination. All control-group animals died on the 8th day, and all DNA & DNA-group animals died on the 9th day after infection with the ectromelia virus. The lack of complete protection can be explained by the use of extremely high doses of the ectromelia virus, which is heterologous to VACV.

## DISCUSSION

Variolation, i.e. intradermal injection of infectious material from smallpox patients to healthy people, was the first method used to protect people from devastating epidemics of smallpox. The disease induced thus had a short incubation period and was relatively mild compared to conventional human-to-human respiratory transmission of the virus. The mortality caused by the inoculation was 0.5–2% as opposed to the 20–30% observed during variola virus epidemics [20]. Discovery of the possibility of human vaccination by inoculation with the cowpox virus and later with the vaccinia virus resulted in a significantly lower risk of severe adverse reactions. In the second half of the XXth century, when VACV was used for immunization, mortality was 1–25 per 1 million vaccinated people [21]. In the case of this vaccination, the risk group included primarily people with immunodeficiency, such as transplant patients, HIV-infected patients, individuals taking immunosuppressive drugs, and others. In this regard, modified vaccines with improved safety characteristics were developed based on VACV. For example, late in the XXth century, Russian researchers developed a live vaccine based on the recombinant strain LIVP VACV, which was tested on humans [22].

To date, there has been no mass vaccination against smallpox. However, there are categories of people who are at risk of becoming infected with smallpox or other pathogenic orthopoxviruses based on their professional occupation. These categories comprise the risk group, and they should undergo obligatory vaccination against smallpox. First, this concerns personnel involved in epidemiological surveillance, the medical staff of infectious departments at hospitals, and employees of virology laboratories dealing with orthopoxviruses. In the case of smallpox outbreaks (e.g., as a result of a bioterrorist attack), all inhabitants of a region must be vaccinated. The conventional first-generation smallpox vaccine based on the LIVP strain, which is currently used for vaccination, has a lot of contraindications and can cause complications with varying severity. It is worth noting that it is somewhat difficult to demonstrate protective immunity against smallpox induced by vaccination of new preventive medication, since the smallpox has been eliminated, and it is impossible to test the efficacy of these vaccines against the natural disease in the absence of epidemics.

Previously, we implemented two independent approaches to the development of safe vaccines against human orthopoxvirus infections. We developed a highly attenuated variant of the vaccinia virus, VACΔ6, with targeted knockdown of six genes, and a polyvalent DNA vaccine based on five antigens of the variola virus. Independent experiments demonstrated that triple immunization with a DNA vaccine and double immunization with VACΔ6 provide protection to mice against a lethal dose (10 LD_50_) of the ectromelia virus, which is highly pathogenic to mice [10, 12].

In this study, we compared the immune response developed against orthopoxvirus using various immunization protocols with a DNA vaccine and VACΔ6. The product of the *A35R *gene, one of the six genes deleted in the recombinant variant VACΔ6, reduces the antigen presentation by the class II major histocompatibility complex. Therefore, the VACΔ6 strain induces a higher level of VACV-neutralizing antibodies than the parental clone LIVP, and it is more effective in protecting animals from ECTV infection at a dose of 150 LD_50_. Combined immunization with a DNA vaccine and a recombinant VACΔ6 variant leads to a lower level of neutralizing antibodies compared to double immunization with VACΔ6. However, it provides the same level of protection. Apparently, this can be attributed to the fact that the DNA vaccine better induces the cell component of the immune response during primary immunization, which is also required for effective orthopoxvirus elimination from the organism [23, 24].

## CONCLUSION

In this study, we used a heterologous immunization strategy to enhance the effectiveness of smallpox vaccination, where the immune system was primed using a polyvalent DNA-vaccine based on five VARV genes, and an attenuated version VACΔ6 was used for subsequent booster vaccination. The level of protection induced this way was the same as that in the option with double immunization using a VACΔ6 strain and superior to that induced by double immunization with the LIVP VACV strain used in the Russian Federation for human vaccination. The proposed immunization protocols can be used to develop safe vaccination strategies against smallpox and other human orthopoxvirus infections. DNA vaccination, followed by vaccination with live-attenuated virus VACΔ6 can be considered as advantageous in terms of safety. It should be noted that the double vaccination protocol is not optimal for emergency prevention of smallpox. In this case, single-dose administration of the conventional smallpox vaccine based of the LIVP VACV strain is advisable.

## Abbreviations

CPXV: cowpox virus
ECTV: ectromelia virus
LD50: 50% lethal dose
LIVP: L-IPV vaccinia virus strain
MPXV: monkeypox virus
PCR: polymerase chain reaction
PFU: plaque forming unit
VACV: vaccinia virus
VARV: variola virus
